# CT Urography Findings of Upper Urinary Tract Carcinoma and Its Mimickers: A Pictorial Review

**DOI:** 10.3390/medicina56120705

**Published:** 2020-12-17

**Authors:** Paola Martingano, Marco F. M. Cavallaro, Alessandro M. Bozzato, Elisa Baratella, Maria A. Cova

**Affiliations:** 1 Department of Radiology, ASUGI, Cattinara Hospital, 34139 Trieste, Italy; 2 Department of Radiology, ASUGI, Maggiore Hospital, 34129 Trieste, Italy; mrc.cavallaro@virgilio.it; 3 Department of Radiology, University of Trieste, ASUGI, Cattinara Hospital, 34139 Trieste, Italy; alessandroj.bozzato@gmail.com (A.M.B.); elisa.baratella@gmail.com (E.B.); m.cova@fmc.units.it (M.A.C.)

**Keywords:** CT urography, urinary tract imaging, urinary tract tumor, urothelial carcinoma, upper tract urothelial carcinoma, benign urinary tract lesions, urinary tract inflammatory disease

## Abstract

Urothelial carcinoma (UC) is the fourth most frequent tumor in Western countries and upper tract urothelial carcinoma (UTUC), affecting pyelocaliceal cavities and ureter, accounts for 5–10% of all UCs. Computed tomography urography (CTU) is now considered the imaging modality of choice for diagnosis and staging of UTUC, guiding disease management. Although its specificity is very high, both benign and malignant diseases could mimic UTUCs and therefore have to be well-known to avoid misdiagnosis. We describe CTU findings of upper urinary tract carcinoma, features that influence disease management, and possible differential diagnosis.

## 1. Introduction

Urothelial carcinoma (UC) is the fourth most frequent malignancy in Western countries, with a peak incidence in elderly (70 to 90-year-olds) and a three-fold man prevalence. Upper tract urothelial carcinoma (UTUC) accounts for 5–10% of all UCs [1,2], with double the incidence of pyelocaliceal tumors compared to ureter location. UTUCs are often associated with concomitant or recurrent bladder cancer, while the contralateral urinary tract is implicated only in 2–6% of tumors. Many risk factors have been identified in the development of UCs, in particular tobacco smoking, some herb consumption, and aromatic amines occupational exposure [3].

Local symptoms of UTUCs, represented by flank pain or lumbar mass, are usually due to advanced disease, while systemic symptoms like fatigue, fever, and weight loss are related to metastatic progression. Hematuria, either microscopic or visible, is the most common and early symptom of UC, and requires prompt investigation. In patient with positive cytology but negative bladder cystoscopy, a UTUC is highly suspected, and so an upper tract evaluation is needed. Flexible ureteroscopy allows direct visualization of ureter, renal pelvis, and collecting system, with the possibility of direct biopsy or aspiration cytology, but it is considered quite invasive. Among imaging techniques, computed tomography urography (CTU) offers the highest accuracy in UTUC diagnosis, with a pooled sensitivity of 92% and a pooled specificity of 95% [3,4]. Other imaging modalities, like magnetic resonance urography (MRU) have been tested, but have been proven to be inferior. In particular, the direct comparison of MRU and CTU showed a sensibility of 82.8–86.2% and a specificity of 83.1–83.3% for MRU compared to 96.6% and 87–91.5% of CTU, respectively [5], so present guidelines indicate CTU as the imaging of choice in the work up of hematuria [3].

## 2. CTU Technique

**Figure 1:**
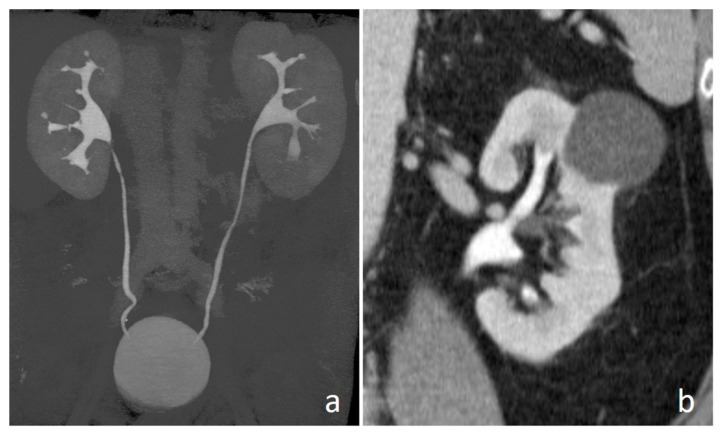
Computed tomography urography (CTU): MIP (maximum intensity projection) (**a**) and coronal (**b**) images. Furosemide administration allows distention of the entire urinary tract, without blooming artifacts due to high attenuation of intraluminal contrast medium; the split-bolus technique allows simultaneous luminal and parenchymal assessment.

CT Urography has to be tailored to clinical indication and could be performed with different protocols, including at least an excretory phase [6]. An unenhanced phase is used to detect stones, calcifications, hemorrhages, clots, and to measure the attenuation coefficients of the renal and urothelial masses [7,8,9]. A corticomedullary phase, occurring between 30 and 40 s after contrast medium administration, is used to evaluate suspected vascular abnormalities or arterial enhancement [9,10], while a nephrographic phase, acquired 90–110 s after contrast medium administration, improves detection and characterization of renal lesions [9,11]. The excretory phase, obtained 8–12 min after contrast agent administration, assesses the abnormalities of the urothelium with the distension and the opacification of the collecting systems, ureters and bladder [8,9,10,11,12]. However, the main limitation of a multiphasic protocols is the high radiation exposure, ranging from 25–35 mSv [6]. To reduce radiation dose, a split-bolus injection of contrast medium could be used, obtaining a single combined nephrographic-excretory phase, lowering the effective dose to 17.5 mSv [13]. To improve the distension and opacification of the collecting system, different ancillary maneuvers can be employed, the most common being intravenous saline hydration and low-dose furosemide injection (Figure 1) [6].

## 3. Imaging Findings

**Figure 2:**
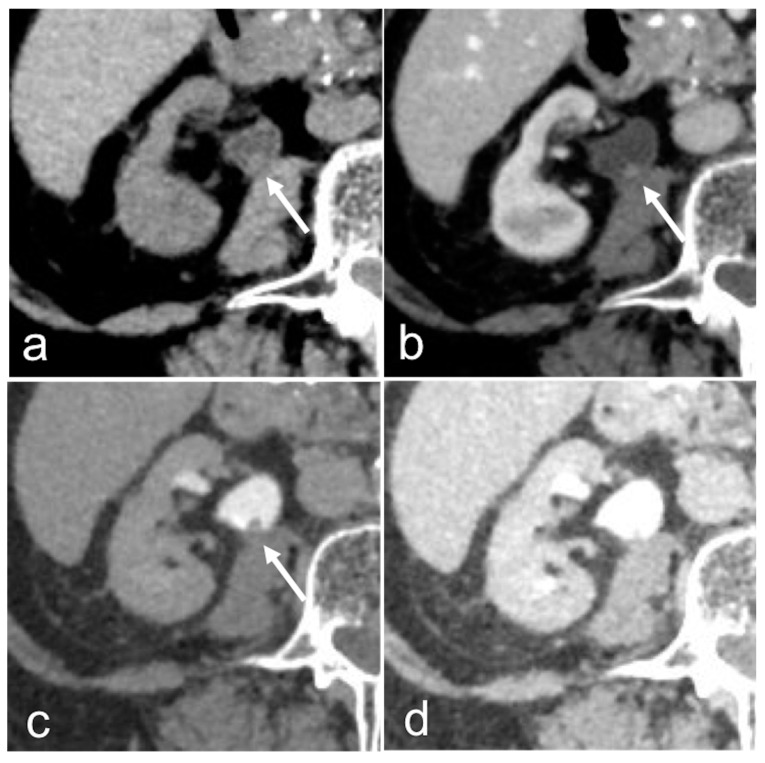
Small renal pelvis UTUC: a soft tissue lesion on unenhanced scan (arrow in (**a**)), shows homogeneous enhancement in the cortico-medullary phase (arrow in (**b**)) and appears as a filling defect in the nephrographic-excretory phase using a wide window setting (arrow in (**c**)). With wrong imaging settings, it results hardly recognizable (**d**).

Upper tract urothelial cell carcinomas appear on unenhanced phase as soft tissue masses, with lower density than renal calculi, except for indinavir ones [9,14,15,16]; intralesional and superficial calcifications can be seen and may appear granular, linear, or punctate [14,17,18,19,20]. After contrast medium administration, UTUCs show early enhancement, unlike non-enhancing clots, while in the excretory phase, they appear as filling defects or luminal narrowing in the urinary tract, best appreciated using a wide window setting, because small tumors may be concealed by the high attenuation of surrounding contrast medium (Figure 2) [9,14,15,16].

**Figure 3:**
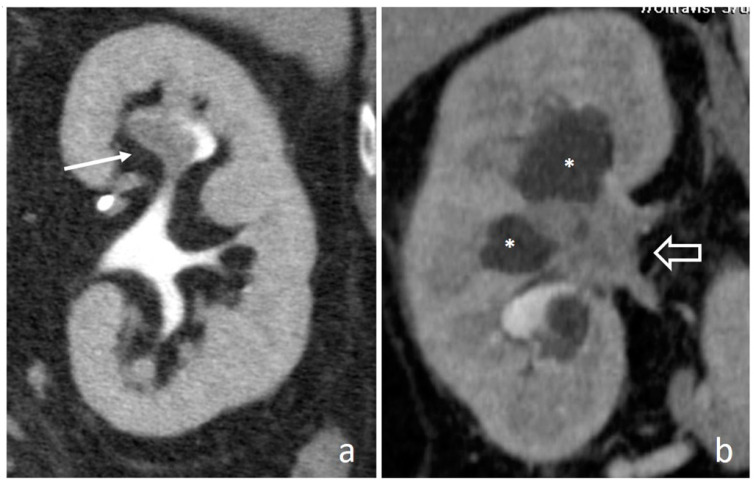
Pyelocaliceal papillary tumors: coronal nephrographic-excretory images (**a**,**b**). Solid tissue enlarges and obscures the superior calyx (arrow in (**a**)), the so-called oncocalyx. Solid pelvic-infundibular tissue (empty arrow in (**b**)) causes upstream non-opacified dilation (*), the so-called phantom calyx.

**Figure 4:**
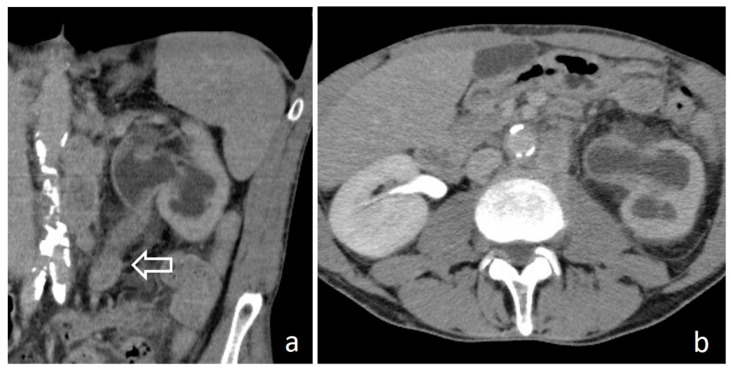
Ureteral papillary upper tract urothelial carcinoma (UTUC): nephrographic-excretory coronal (**a**) and axial (**b**) images. Solid tissue in the lumbar ureter (empty arrow in (**a**)) determines complete occlusion of the urinary tract with hydronephrosis. Note the reduced nephrographic effect and absent excretion of contrast medium in the left kidney.

**Figure 5:**
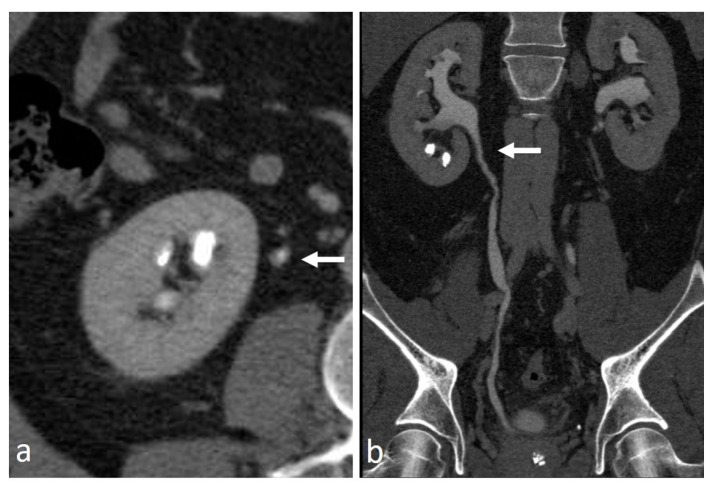
Flat ureteral UTUC: nephrographic-excretory axial (**a**) and CPR—curved planar reconstruction—coronal (**b**) images. An irregular wall thickening (arrows), without lumen narrowing, extends in the upper ureter. Urinary stones are visible in inferior calyces.

**Figure 6:**
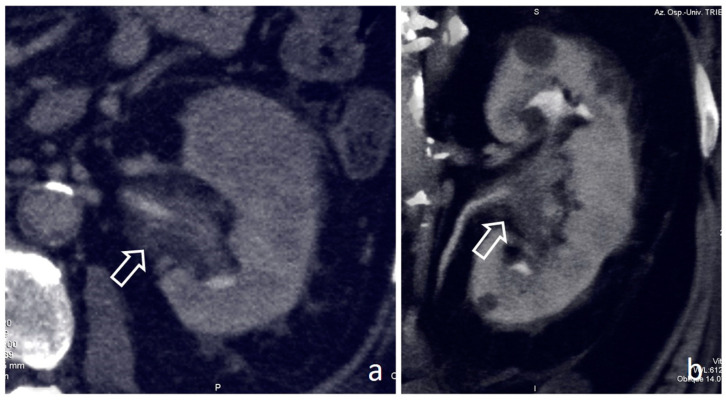
Pyelocaliceal infiltrative UTUC: nephrographic-excretory axial (**a**) and coronal (**b**) images. Extensive pyelocaliceal wall thickening with lumen narrowing causes diffused attenuation of the renal sinus fat due to tumoral infiltration (empty arrows).

**Figure 7:**
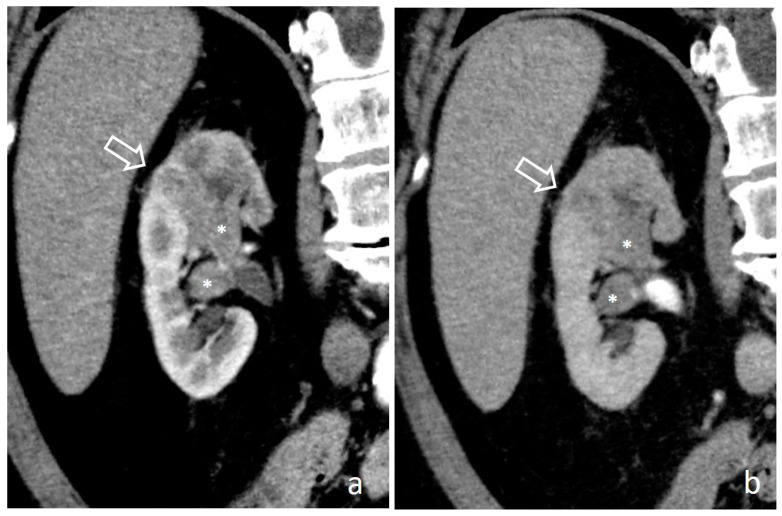
Caliceal infiltrative UTUC: coronal cortico-medullary (**a**) and nephrographic-excretory (**b**) images. Solid tissue occupies upper and medium calyces (*); the direct infiltration of kidney parenchyma appears hyper-enhancing compared to medulla in the cortico-medullary phase (arrow in (**a**)) and shows wash out in the nephrographic phase (arrow in (**b**)).

Based on their morphology UTUCs may be differentiated in papillary tumors, flat lesions, and invasive carcinomas. Papillary tumors appear on CTU as single or multiple filling defects in the collecting system, varying from millimetric to broad basis wide tumors occupying the entire lumen [14,16,17]. When tumors involve the caliceal system, a calyx could be entirely missing in the excretory phase due to complete filling by solid tissue, the so-called “oncocalyx”(Figure 3a); calyx dilation and absence of urine opacification, classically described as “phantom calyx”, occur if tumor develops in the infundibular neck determining its amputation (Figure 3b) [15,17,21,22]. When tumors involve the ureter, lumen occlusion is frequent and hydronephrosis can cause hypoperfusion of the renal parenchyma; on CTU, the solid ureteral mass is accompanied by a reduced nephrographic effect and a delayed contrast agent excretion (Figure 4) [16,17]. Flat lesions could manifest as a concentric or eccentric pyelocaliceal or ureteral wall thickening, not invariably causing luminal narrowing (Figure 5). Finally, UTUCs can present an infiltrative pattern with increased attenuation and stranding of periureteral and renal sinus fat (Figure 6). Direct involvement of renal parenchyma may be present in pyelocaliceal malignancy; on CTU, it is easily appreciated in the cortico-medullary phase thanks to early UC enhancement compared to renal medulla, while in the nephrographic phase, tumor tissue hypoenhancement is more difficult to differentiate from reduced nephrographic effect of the compressed adjacent parenchyma (Figure 7). Sometimes, in advanced cases, infiltration causes kidney enlargement, but with the preservation of the organ shape [9,16].

CTU findings help distinguishing organ-confined diseases from advanced ones, but infiltrative tumors are correctly diagnosed only in 67% of cases; this low performance is due to microscopic invasion, not visible at imaging, or to misinterpretation of inflammatory changes as tumor involvement [23]. Therefore, in clinical practice multiple clinical, histological, and imaging parameters are used to stratify tumor risk and its consequent management. According to European Association of Urology guidelines, low-risk tumors, identified by unifocal disease, size < 2 cm, non-invasive aspect on CTU, and low-grade on cytologic or biopsy specimens, could be treated with kidney-sparing surgery (endoscopic ablation and segmental ureteral resection); on the other hand radical nephroureterectomy and systemic chemotherapy are indicated in case of multifocal disease, size > 2 cm, invasive aspect on CTU, concomitant hydronephrosis, high-grade cytology, high grade or variant histology, or previous cystectomy [3].

## 4. Mimickers of UTUC

Many pathological conditions may mimic CTU appearance of UTUC, both benign and malignant. Some CTU features could help differential diagnosis, but they are not always sufficient and so further diagnostic work-up may be required. Among these lesions, hypertrophied papilla, clots, suburothelial hemorrhage, renal papillary necrosis, symmetric wall thickening of the urinary tract due to inflammation or encasement, cystic pyeloureteritis, urogenital tuberculosis, and fibroepithelial polyp are benign lesions that should be considered to avoid an unjustified invasive intervention. On the other hand, renal cell carcinoma and renal lymphoma may be mistaken for UTUC and receive incorrect treatment.

### 4.1. Hypertrophied Papilla

**Figure 8:**
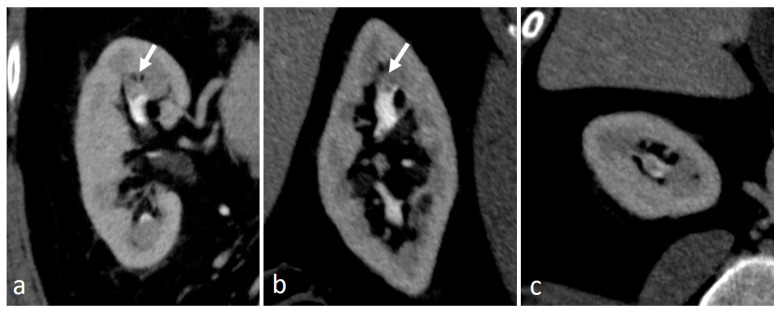
Hypertrophied papilla: nephrographic-excretory coronal (**a**), sagittal (**b**) and axial (**c**) images. A filling defect with smooth margins and regular shape is recognizable in the superior calyx (arrows); the forniceal structure is preserved.

Hypertrophied papilla, a benign anatomical abnormality, causes a pronounced concave impression on surrounding calyx that can mimic a filling defect in the excretory phase. The presence of a smooth contour with preservation of forniceal shape (Figure 8), along with the frequent presence of multiple similar aspects in the same kidney, allows correct diagnosis [24,25].

### 4.2. Blood Clots

**Figure 9:**
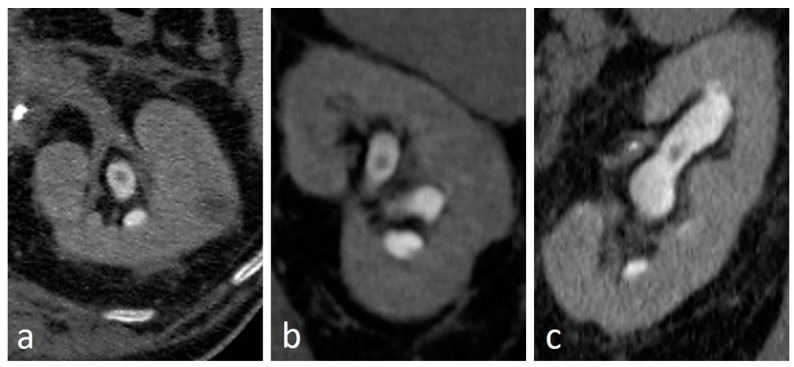
Blood clot: nephrographic-excretory axial (**a**), coronal (**b**) and sagittal (**c**) images. A small filling defect in the superior calyx without contact with the caliceal wall.

Blood clots are usually caused by trauma, neoplasm, renal lithiasis and anticoagulant therapy. They commonly manifest as slightly hyperdense lesions on unenhanced phase, without enhancement after contrast medium administration; in the excretory phase, they can be more easily differentiated from UTUC, because commonly entirely surrounded by opacified urine (Figure 9). In doubtful cases a further CT scan performed after changing patient position demonstrates a shift of the suspicious filling defect [7,9,12].

### 4.3. Suburothelial Hemorrhage

**Figure 10:**
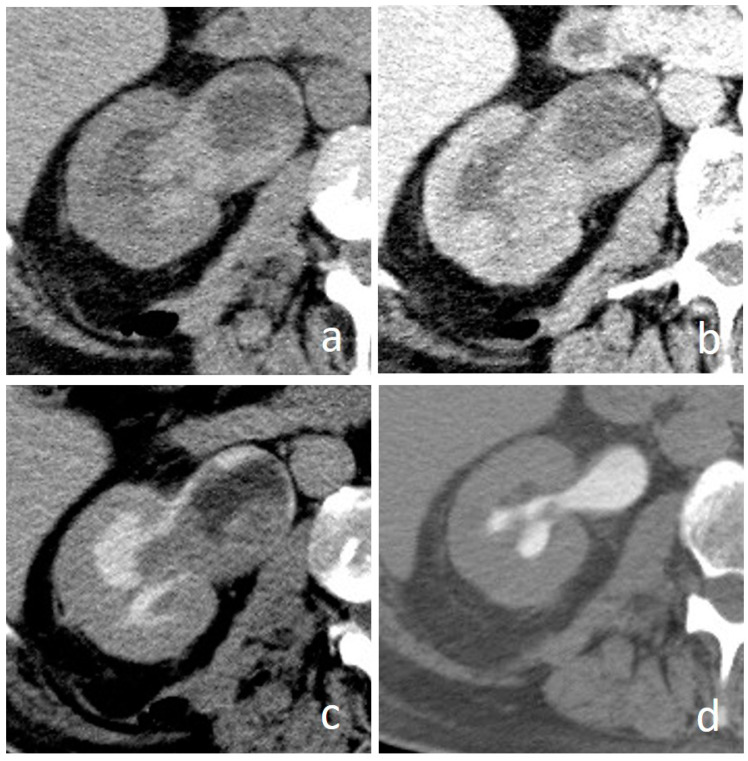
Suburothelial hemorrhage: axial images. In the unenhanced scan (**a**) diffuse hyperdense urothelial wall thickening is clearly visible. In the nephrographic (**b**) and excretory (**c**) phases wall thickening is hardly differentiable from a UTUC. Complete disease resolution on follow up CTU (**d**) confirmed the benign nature of previous extensive urothelial thickening.

Suburothelial hemorrhage is a rare condition with diffuse renal sinus wall thickening due to intraparietal bleeding, presenting with flank pain and hematuria. This condition is related to bleeding diatheses such as anticoagulant treatment and hematological disorders. In the contrast enhanced and excretory phases, the renal pelvic wall shows diffuse soft tissue thickening with lumen narrowing, easily mistaken for a UTUC. The unenhanced phase allows the correct differential diagnosis demonstrating hyperdensity of the urinary tract wall due to hemorrhagic components (Figure 10) [26,27].

### 4.4. Renal Papillary Necrosis

**Figure 11:**
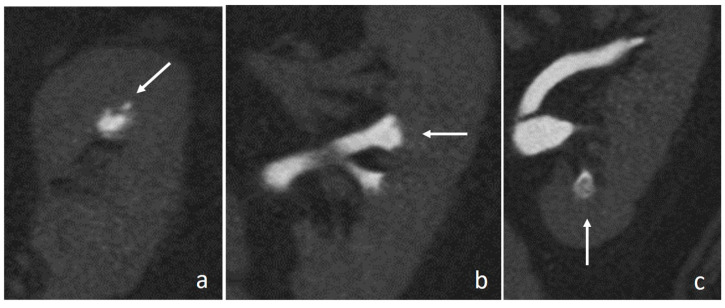
Renal papillary necrosis: excretory coronal images. The central erosion of a papilla appears as a focal spot of opacified urine inside renal medulla, with a teardrop shape (arrow in (**a**)) and a ball-on-tee shape (arrow in (**b**)); in the end stage necrotic papilla creates a filling defect in the excavated calyx, the signet ring sign (arrow in (**c**)).

Renal papillary necrosis (RPN), often presenting with hematuria, is due to an ischemic lesion involving the renal papilla; it is frequently associated with diabetes mellitus, sickle cell anemia, analgesic abuse and urinary tract infections [11]. RPN is recognizable only in the excretory phase, presenting as central erosion of the papilla filled with opacified urine, the teardrop-shaped cavity (Figure 11a) or the “ball-on-tee” sign (Figure 11b), or as erosion of the calyceal fornices filled with opacified urine, the “lobster-claw calyx”. In the end stage, the sloughed necrotic papilla appears as a non-enhancing filling defect surrounded by contrast medium in the excavated calyx, the signet ring sing (Figure 11c) [14,25]. Beyond the possible presentation, RPN determines caliceal irregularity extending proximal to interpapillary line, with opacified urine located deep into the medulla, while calyceal irregularities due to UTUC extend toward renal pelvis [19].

### 4.5. Inflammation

**Figure 12:**
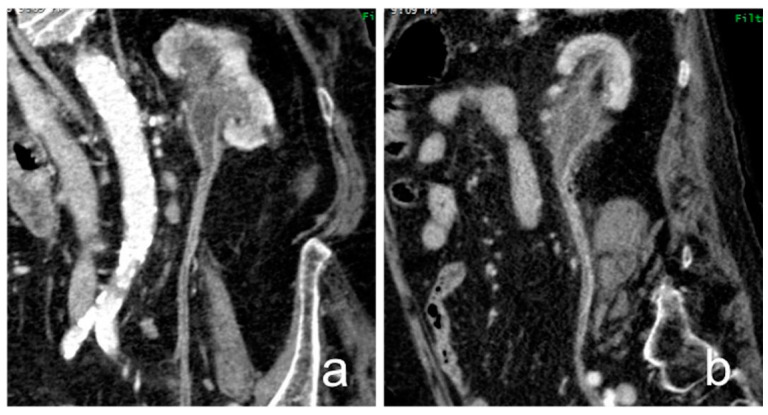
Inflammatory disease: CPR cortico-medullary images in corona (**a**) and sagittal (**b**) planes. Diffuse urothelial thickening with homogenous enhancement associated to haziness of sinus fat and mild hydronephrosis.

**Figure 13:**
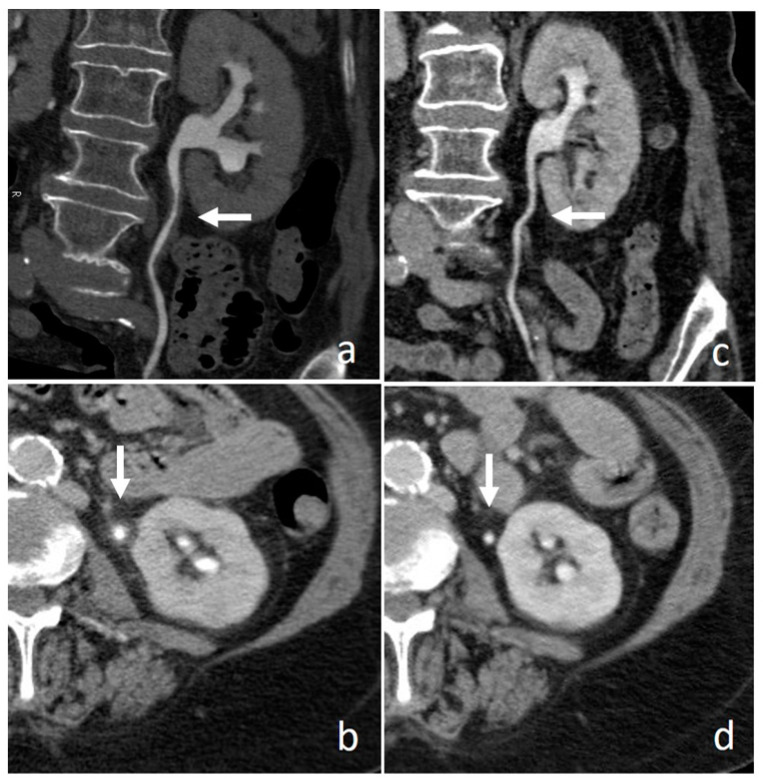
Inflammatory disease. Nephrographic-excretory CPR (**a**,**c**) and axial (**b**,**d**) images. A short ureteral wall thickening, with smooth margins and symmetric appearance, causes mild lumen stricture (arrows in (**a**,**b**)); clinical and laboratory data are indicative of an inflammatory disease. The follow up CTU performed 3 months later (**c**,**d**), demonstrates a complete resolution, confirming the benign nature.

Pyeloureteral wall thickening with increased attenuation of the surrounding fat is not a specific sign of UTUC but may be present also with urothelial inflammatory diseases. Differential diagnosis is not always possible on the basis of imaging alone, and so clinical, laboratory, and, sometimes, pathological findings have to be considered to decide proper management. The involvement of a longer part of the urinary tract is usually related to a benign disease, while UTUC is often associated with a shorter segment localization [7,12]. Other signs indicative of a benign disease are circumferential symmetric wall thickening, with diffuse urothelial enhancement, smooth margins, and symmetric and homogeneous attenuation of the surrounding fat (Figure 12); moreover, in doubtful cases, a follow-up CT may be useful to confirm benign nature of urothelial thickening (Figure 13) [28,29].

### 4.6. Retroperitoneal Fibrosis

**Figure 14:**
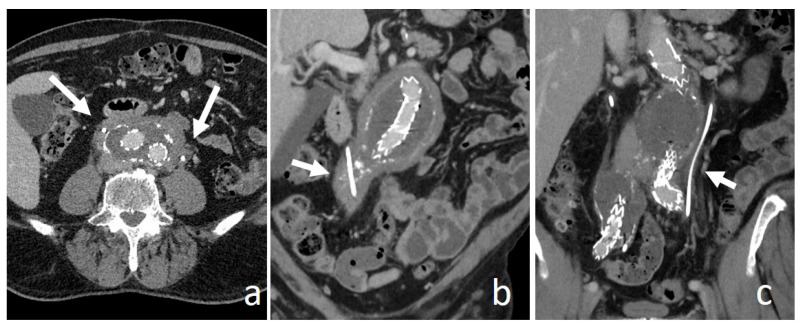
Retroperitoneal fibrosis. Nephrographic axial (**a**) and coronal (**b**,**c**) images. Low enhancing solid tissue surround an aneurismatic abdominal aorta, causing encasement and medial deviation with narrowing of both ureters (arrows), already treated with ureteral stents.

Retroperitoneal fibrosis (RF) is a fibro-inflammatory disease characterized by the development of fibrotic tissue in the retroperitoneum; it could be idiopathic, associated to abdominal aortic aneurism, or secondary to other diseases. RF can cause ureteral encasement with obstruction and hydronephrosis. On CT it appears as enhancing solid tissue, generally isodense to muscle, that develops around the abdominal aorta, causing obscuration of fat planes, and medial displacement of the ureters (Figure 14) [29].

### 4.7. Pyeloureteritis Cystica

**Figure 15:**
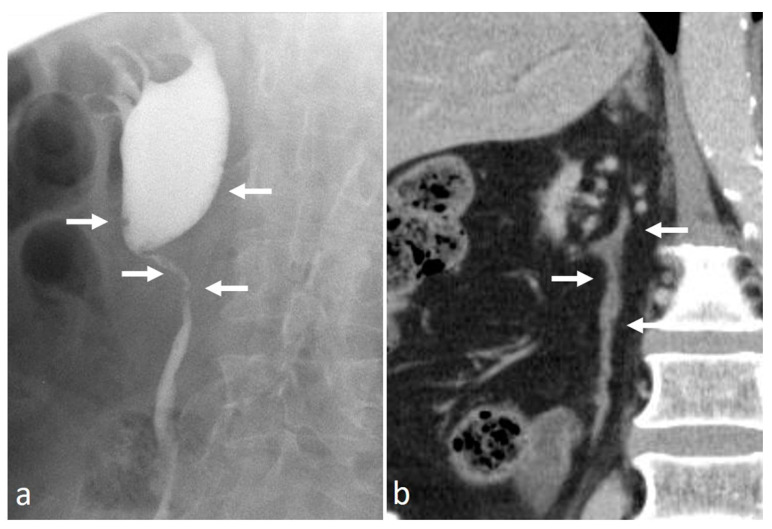
Pyeloureteritis cystica: ascending pyelography (**a**) and CT nephrographic coronal CT (**b**) images. Multiple small and regular filling defects are recognizable in the renal pelvis and proximal ureter (arrows in (**a**)), which correspond to tiny hypodense lesions inside a diffuse enhancing urothelial wall thickening (arrows in (**b**)).

Pyeloureteritis cystica is a benign disease due to chronic inflammation of the urinary tract, characterized by multiple subepithelial cysts, arising from degeneration of von Brunn’s nests. On unenhanced phase, the measurement of the attenuation coefficients of the cysts is usually not possible due to their tiny dimensions and, on excretory phase, they appear as multiple millimetric filling defects, often not detectable on nephrographic phase (Figure 15). The differential diagnosis between pyeloureteritis cystica and multifocal UTUC may be difficult. Nevertheless, the latter is usually characterized by presence of fewer and more inhomogeneous lesions [7,12,29,30].

### 4.8. Urogenital Tuberculosis

**Figure 16:**
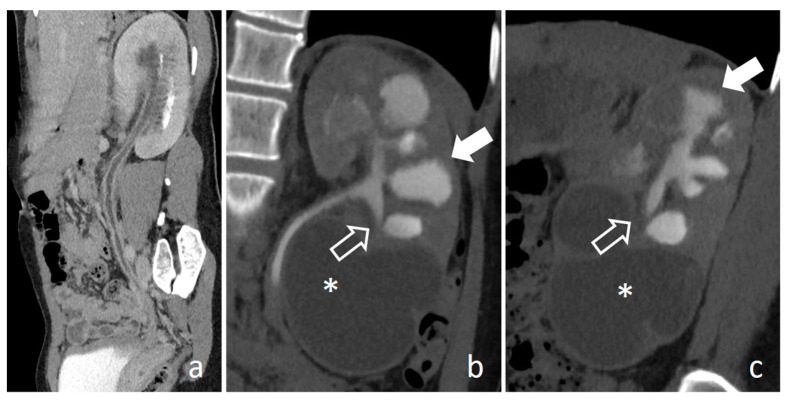
Urogenital tuberculosis: nephrographic-excretory CPR (**a**), coronal (**b**) and sagittal (**c**) images. Widespread enhancing urothelial wall thickening affects renal pelvis and the entire ureter (**a**). Multiple caliceal erosions with complete expulsion of necrotic papilla are recognizable in the upper and medium part of the kidney (full arrows), the inferior calyx presents a rose-thorn shape (empty arrows), while the lower pole is replaced by a large fluid cavity (*).

Urogenital tuberculosis (UGTB) accounts for almost one third of extrapulmonary TB cases, and 3% of patients with TB develop renal manifestations [31]. In the early stage of UGTB, the most typical imaging findings are papillitis, papillary necrosis and caliceal deformity, the so-called moth-eaten calyx [12,22,32]. During progression of the disease, granulomas coalesce and form the tuberculomas, i.e., caseous necrotic lesions with hypodense non-enhancing center, in direct communication with calyces [12]. At this stage, a fibrotic response develops, causing strictures with caliectasis, or amputation of the calyces, both characterized by a typical “rose-thorn” shape [12,22,31]. Moreover, pelvic stricture and retraction may be seen along with an irregular thickening of the pelvis and the ureter [22,33]. The differential diagnosis between UGTB and UTUC may be difficult. However, UGTB must be highly suspected when multiple different caliceal deformities coexist in the same patient (Figure 16) [28].

### 4.9. Fibroepithelial Polyp

**Figure 17:**
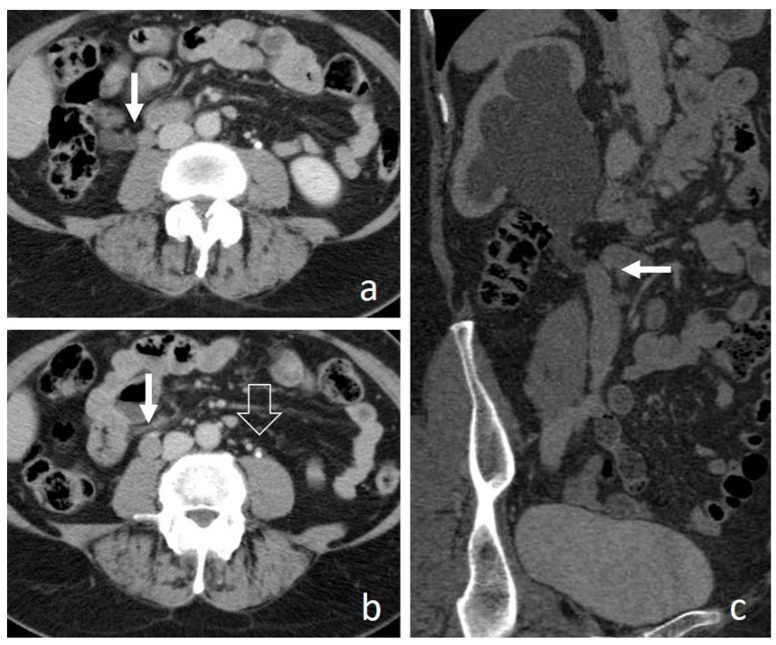
Fibroepithelial polyp: nephrographic-excretory axial (**a**,**b**) and CPR (**c**) images. A smooth-margin mass develops inside the ureter without associated wall thickening (arrows). Note complete lumen occlusion with upstream dilation and absent contrast excretion, by comparison with the contralateral normal ureter (empty arrow). When typical aspects are absent, differential diagnosis with UTUC is difficult.

A fibroepithelial polyp (FP) is a benign ureteral lesion, consisting of a fibrovascular stroma, covered by a layer of urothelium. It usually presents with hematuria or flank pain, and with a CTU aspect of a solid mass inside renal pelvis or ureter, so the differential diagnosis with a papillary UTUC is challenging (Figure 17). The correct diagnosis could be supposed only in the presence of the typical appearance of a long pedunculated mass with smooth margins, entirely surrounded by contrast medium in the excretory phase, except for the attachment site of the stalk [12,28,29].

### 4.10. Renal Cell Carcinoma

**Figure 18:**
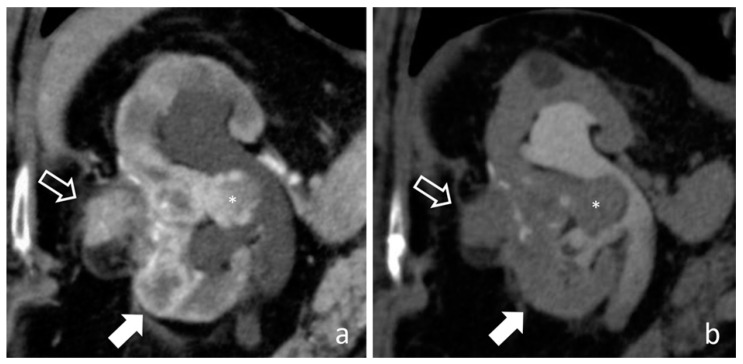
Recurrent RCC: cortico-medullary (**a**) and excretory (**b**) coronal images. A polilobulated lesion with inhomogeneous early contrast enhancement (full arrows) develops inside the renal parenchyma and extend in renal pelvis (*). The organ shape is altered and an exophytic mass is recognizable in the site of previous local surgery (empty arrows).

Renal Cell Carcinoma (RCC) typically appears as an expansive mass with well-defined margins and it is easily differentiated from urothelial carcinomas. Nevertheless, infiltrative RCCs could sometimes be difficult to distinguish from pyelocaliceal infiltrative UTUCs. Both malignancies show early contrast enhancement. However, the reniform shape is usually preserved in infiltrative tumors of the excretory tract, whereas a contour expansion can be seen in RCCs (Figure 18) [14,16,34].

### 4.11. Renal Lymphoma

**Figure 19:**
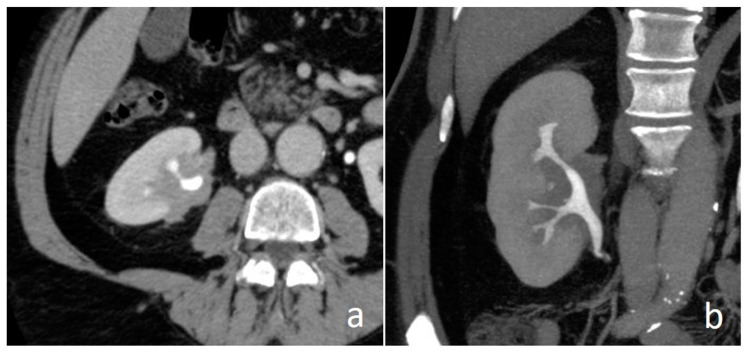
Renal sinus lymphoma: nephrographic-excretory axial (**a**) and MIP (**b**) images. Hypovascular and homogenous solid tissue diffusively involves renal sinus fat without distortion of pyelocaliceal system.

Extranodal spread of lymphoma often involves the urinary tract, but primary renal lymphoma without systemic disease is quite rare. Lymphomatous tissue could develop from renal capsule or perinephric and hilar fat, determining a vascular encasement and renal sinus involvement. It presents as a homogeneous soft-tissue lesion on non-enhanced CT with poor enhancement after contrast injection due to its hypovascularity, helping differential diagnosis with typical hypervascular RCCs and UTUCs. Lymphoma of the renal sinus could mimic the infiltrative pattern of UTUC, but lymphomatous tissue, due to its pliable nature, generally do not alter urinary tract shape nor causes hydronephrosis (Figure 19) [16,35,36].

## 5. Conclusions

Computed tomography urography is a very useful tool in diagnostic workup of suspected upper urinary tract carcinoma. Some CTU features help to differentiate low-risk tumors from high-risk ones, leading to proper management. However, imaging findings could be nonspecific, and only the knowledge of possible UTUC mimickers guides a correct differential diagnosis, avoiding mistreatment.

## Abbreviations

CTU	Computed Tomography Urography;
FP	fibroepithelial polyp;
MIP	maximum intensity projection;
MPR	multiplanar reconstruction;
MRU	magnetic resonance urography;
RCC	renal cell carcinoma;
RF	retroperitoneal fibrosis;
RNP	renal papillary necrosis;
UC	urothelial carcinoma;
UGTB	urogenital tuberculosis;
UTUC	upper tract urothelial carcinoma
